# CREdb: A comprehensive database of Cis-Regulatory Elements and their activity in human cells and tissues

**DOI:** 10.1186/s13072-024-00545-7

**Published:** 2024-07-16

**Authors:** Chris Hartl, Jiali Zhuang, Aaron Tyler, Bing Zhou, Emily Wong, David Merberg, Brad Farrell, Chris DeBoever, Julie Bryant, Dorothée Diogo

**Affiliations:** 1 Rancho BioSciences LLC, San Diego, California USA; 2 Genetics and Systems Biology, Takeda Development Center Americas, Inc, San Diego, CA 92121 USA; 3 Genetics and Systems Biology, Takeda Development Center Americas, Inc, Cambridge, MA 02139 USA; 4 Present Address: Data Science and Operations, Vir Biotechnology Inc, San Francisco, CA 94158 USA

## Abstract

**Background:**

Cis-regulatory elements (CREs) play a pivotal role in gene expression regulation, allowing cells to serve diverse functions and respond to external stimuli. Understanding CREs is essential for personalized medicine and disease research, as an increasing number of genetic variants associated with phenotypes and diseases overlap with CREs. However, existing databases often focus on subsets of regulatory elements and present each identified instance of element individually, confounding the effort to obtain a comprehensive view. To address this gap, we have created CREdb, a comprehensive database with over 10 million human regulatory elements across 1,058 cell types and 315 tissues harmonized from different data sources. We curated and aligned the cell types and tissues to standard ontologies for efficient data query.

**Results:**

Data from 11 sources were curated and mapped to standard ontological terms. 11,223,434 combined elements are present in the final database, and these were merged into 5,666,240 consensus elements representing the combined ranges of the individual elements informed by their overlap. Each consensus element contains curated metadata including the number of elements supporting it and a hash linking to the source databases. The inferred activity of each consensus element in various cell-type and tissue context is also provided. Examples presented here show the potential utility of CREdb in annotating non-coding genetic variants and informing chromatin accessibility profiling analysis.

**Conclusions:**

We developed CREdb, a comprehensive database of CREs, to simplify the analysis of CREs by providing a unified framework for researchers. CREdb compiles consensus ranges for each element by integrating the information from all instances identified across various source databases. This unified database facilitates the functional annotation of non-coding genetic variants and complements chromatin accessibility profiling analysis. CREdb will serve as an important resource in expanding our knowledge of the epigenome and its role in human diseases.

**Supplementary Information:**

The online version contains supplementary material available at 10.1186/s13072-024-00545-7.

## Background

In eukaryotic organisms, cis-regulatory elements (CREs) such as enhancers, promoters, silencers, and transcription factor binding sites and their selective activation provide a flexible mechanism of transcriptional regulation, allowing cells with the same genetic code to serve diverse roles throughout the body and respond to external stimuli such as stress [1] and drugs [2]. These regulatory elements control when genes are expressed and can play a pivotal role in drug development and therapeutic innovation, especially as we aim to apply personalized medicine principles to ensure that we are providing not just the best drug for a specific condition, but the best drug for the patient [2]. In addition, genetic variants in CREs have been found to account for some of the common disease heritability [3], and the effects of several trait-associated genetic variants identified through genome-wide association studies (GWAS) are mediated through their activity as expression modulators [4], making their understanding critical for deciphering these traits and conditions.

High throughput sequencing technologies such as Assay for Transposase-Accessible Chromatin using sequencing (ATAC-seq) and Chromatin Immunoprecipitation Sequencing (ChIP-seq) enable researchers to discover the activities of regulatory elements in various cellular contexts. High-quality databases containing annotated known regulatory elements would aid the characterization of the epigenetic signatures identified by these assays. Many such databases exist, including ENCODE [5], ENdb [6], and RefSeq [7], but most focus on a subset of regulatory elements and none define the relationship between all these elements to provide a comprehensive picture of the epigenetic regulation they enact. An increasing number of genetic variants found to associate with important human phenotypes and traits in GWASs fall within the intergenic or intronic regions and do not alter protein amino acid sequences [4]. The prevailing theory is that many of those variants may alter CRE sequences and therefore affect gene expression levels. The effort to elucidate the effects of genetic variants on CREs is hindered by the lack of a centralized database that compiles and annotates all known CREs. Additionally, single-cell/single-nucleus ATAC-seq has been increasingly adopted to study CREs and their regulatory roles at the cell type resolution. Although it has shown great success in uncovering CREs that are present in a subset of the cells, correctly annotating and classifying those CREs entails a comprehensive and well-defined resource containing known CREs.

To address this gap, we have combined regulatory element data from 11 sources into a database with over 10 million human regulatory elements across 1,058 cell types and 315 tissues. These data were aligned to standard ontological terms and structured in database-compatible tables. Consensus tables were generated from data of all available datasets to provide consensus calls for elements, their activity within each biosample in the database, and all elements with genomic overlap that may represent similar activity. This database provides a comprehensive resource for evaluating epigenetic signatures and simplifies the process of evaluating all elements simultaneously.

## Results

### Data curation

**Fig. 1:**
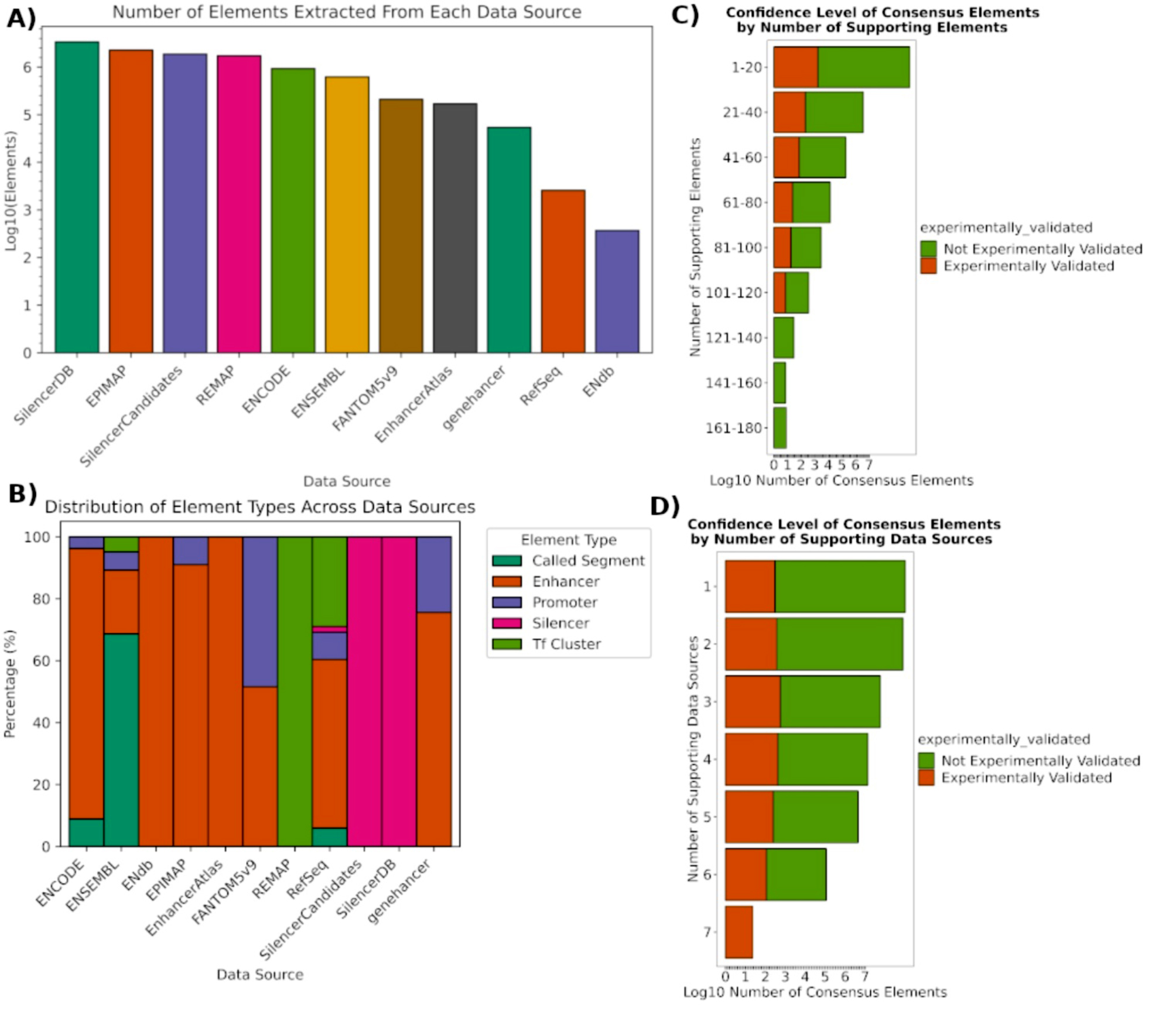
CRE Distribution by source and confidence level. **(A)** Log10 number of CREs derived from each data source. **(B)** Percentage of CREs from each data source belonging to each type of CRE. **(C)** Log10 number of consensus CREs grouped by the number of elements supporting them, as well as how many within these groups are experimentally validated. Of note, most enhancers were experimentally validated. **(D)** Log10 number of consensus CREs grouped by the number of data sources that provided supporting elements to them, as well as how many within these groups are experimentally validated. Of note, mostly enhancers were experimentally validated

Data were extracted from the eleven data sources (see Supplemental Methods) including 3,326,498 enhancers, 390,522 promoters, 5,236,880 silencers, 1,760,581 TF clusters, and 508,953 other elements along with their relevant metadata. The distribution of the extracted elements by source and element type can be found in Fig. 1A and B. A total of 21,328,433 gene-CRE pairs were included as well as 44,861 enhancer-promoter interactions. To provide cellular and physiological contexts to CRE activities, CREdb defined and cataloged 3,675 raw biosample terms, representing 1,058 cell types, 315 tissue types, and 202 diseases.

### Combined and consensus results

**Fig. 2:**
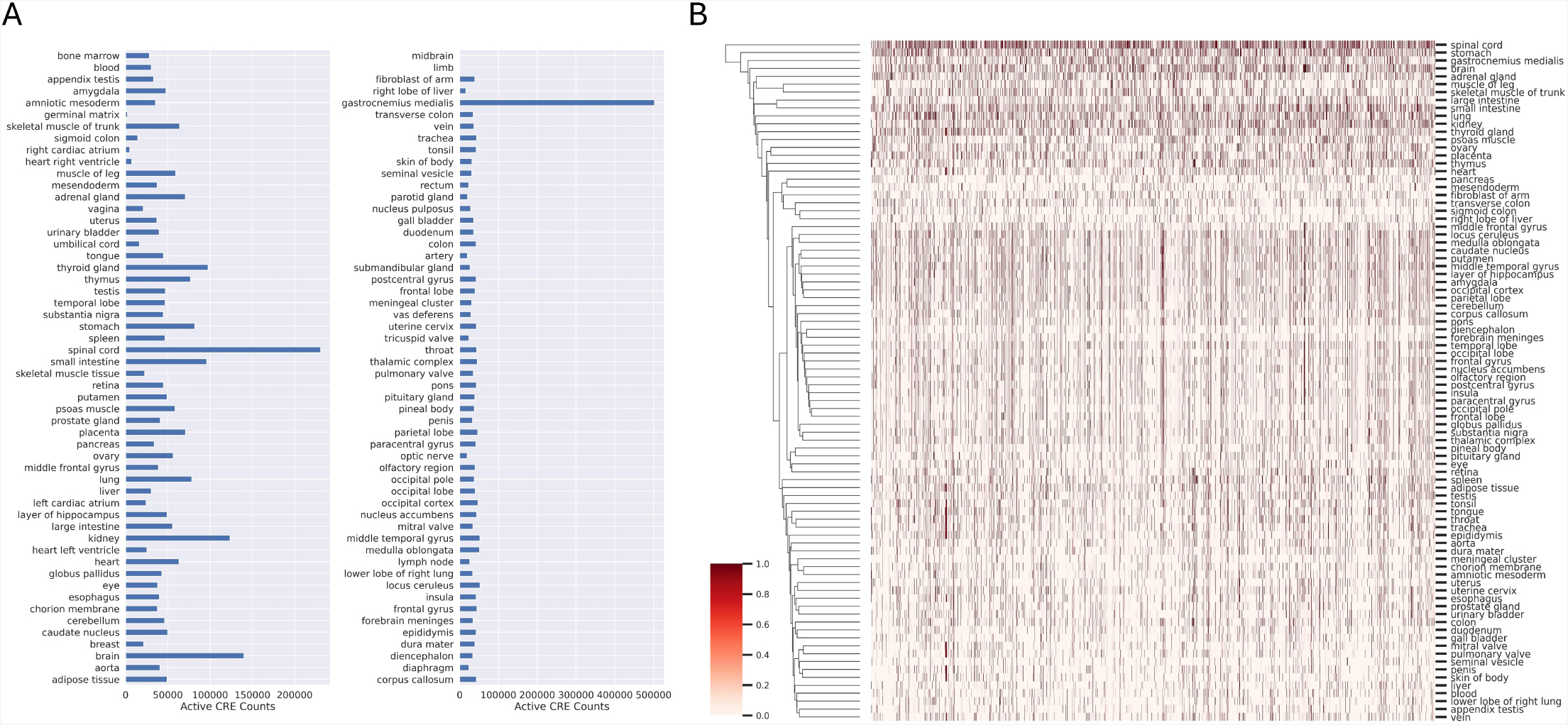
Distribution of elements by tissue source. **(A)** Distribution of active elements by tissue source. **(B)** Hierarchical clustering of tissues based on CRE activity patterns. CREs that are too rare (active in fewer than 3 tissues) or too common (active in more than half of all the tissues) are excluded from the clustering analysis. Active elements are in red, inactive elements in white

A total of 11,223,434 combined elements are present in the final database, and 5,666,240 consensus elements were derived from these to better define the genomic range of each element to facilitate more inclusive detection of these elements from sequencing data. Each consensus element contains metadata including the number of elements supporting this consensus and a hash indicating which of the databases these supporting elements were derived from to assist in gauging the relative strength of these consensus elements in downstream analysis. The data supporting the confidence level in these consensus elements are shown in Fig. 1C and D. A total of 641,405,593 consensus activity rows are present in the final database, defining the activity state of each consensus element in each biosample it was measured in. The distribution of consensus elements by tissue can be found in Fig. 2A. We also perform hierarchical clustering of the tissues based on their CRE usage patterns (Fig. 2B), which confirms that related tissues tend to share similar CRE activities. For instance, different brain regions are clustered together.

### Activity by contact (ABC) results

A total of 19,864,642 candidate enhancers across 131 biosamples were extracted from the data provided by Nasser et al. [8]. As these data were published after the construction of the database, they were not included in the primary model of CREdb. Instead, they are provided as a separate table with the relevant information needed to align them with the existing elements in CREdb.

### Examples of applying CREdb to facilitate genomic research

**Fig. 3:**
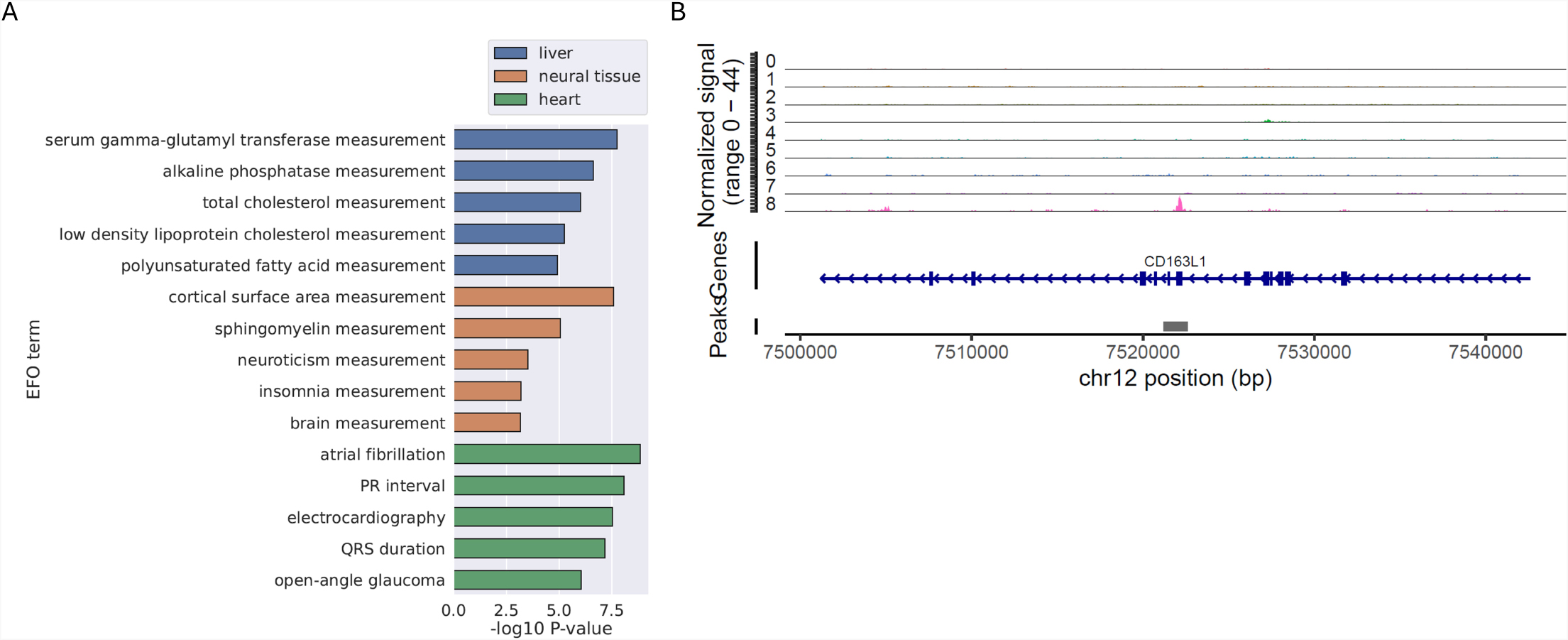
Example Uses of CREdb. **(A)** Top five phenotypes whose significant GWAS hits are enriched in consensus elements active in liver, neural tissue and heart, respectively. **(B)** Example single-nucleus ATAC-Seq analysis utilizing CREdb to identify CREs not detected by standard peak-calling analysis. Each track in the signal profile represents a cell cluster with Cluster8 (shown in pink) representing smooth muscle cells

One potential application of the comprehensive CRE database, as we presented here, is to provide functional context to genomic regions that are found to associate with certain traits or phenotypes. We extracted consensus elements that are active in only one of the three tissues: liver, neural tissue, and heart, and intersected those regions with SNPs that are reported to associate with any of the phenotypes compiled in the GWAS catalog [9]. For each tissue, we then tested the enrichment of each phenotype by comparing the observed and expected number of lead SNPs that fall within the tissue-specific regulatory elements. Figure 3 A shows the top five most enriched phenotypes for each of the three tissues. For liver, the phenotypes whose lead SNPs significantly overlap with liver-specific regulatory elements include liver enzyme levels (serum GGT and alkaline phosphatase) and metabolism (cholesterol and fatty acid levels). For neural-specific regulatory elements, the most enriched phenotypes appear to be related to brain physiology such as cortical surface area, sphingomyelin level, neuroticism, and insomnia. Similarly, atrial fibrillation and electrocardiographic measures are the top phenotypes whose lead SNPs are enriched in heart-specific regulatory elements. This analysis uncovers the link between tissue specific CREs and the physiology and function of the tissue. Additionally, it demonstrates the possibility of using CREs to better annotate and understand the signals derived from association studies. One can also expand beyond the lead variant to include variants in the credible set or the linkage disequilibrium (LD) block, which will likely increase the sensitivity of the analysis.

To explore the utility of CREdb in enhancing single-cell ATAC-seq analysis, we downloaded and analyzed a public single-nucleus ATAC-seq dataset generated from a postmortem human brain (substantia nigra) sample [10]. Among the major cell types identified in this dataset is a smooth-muscle-cell-like population that is characterized by high activity score (measured as promoter and gene body DNA accessibility) for genes such as *TALGN* (encoding Transgelin) and *MYL9 (Myosin regulatory light polypeptide 9)*. We noticed that an open chromatin region specific to this smooth muscle cell population happens to fall within a CREdb consensus element (TAKCRE003692821) which is active only in smooth muscle cell of the brain vasculature (Fig. 3B). Remarkably, this open chromatin region was not identified in the original analysis presumably because it is only present in a small number of cells and could be missed by the peak calling algorithm. This example highlights the potential value of complementing peak calling with a list of pre-annotated CREs in single-cell/single-nucleus ATAC-seq data analysis.

## Conclusions

Cis-regulatory elements from multiple publicly available databases were mapped to standard ontologies to better support their use, combined into consensus elements to increase their sensitivity in detecting the availability of these elements from sequencing results, and compiled into a database made available for public use as CREdb.

CREdb enables researchers to better understand the human genome by providing a broader and more in-depth look at experimental results in the context of known regulatory elements across the current literature. For example, it can aid in annotating GWAS signals in non-coding regions by aligning these signals to consensus elements and identifying in what tissue and cell types those elements were active. In the case where the target genes of the elements are available, it provides a clue as to what genes may play a role in the trait/phenotype in question. It can also be used to identify candidate CRE regions in single-cell ATAC-seq analysis to detect peaks in consensus element regions that might not be detected with a single dataset. Within the rapidly expanding field of single-cell chromatin accessibility sequencing, this should aid in expanding our knowledge of the epigenome and its role in human disease.

Linking a locus associated with a trait of interest to the causal gene is crucial in drug target discovery and prioritization efforts. Conversely, carefully examining all the traits that are associated with a given gene may reveal potential safety concerns and therefore inform safety evaluation for candidate drug targets. CRE-gene linkage information provided in CREdb such as enhancer-gene pairs and activity by contact measurements may complement existing models (for instance, Open Targets Genetics L2G score) in facilitating the nomination of causal genes at GWAS loci. Existing databases of CREs typically focus on one or several types of CREs, making a systematic evaluation of all CRE types cumbersome. Furthermore, each database only contains CREs derived from a limited range of cellular contexts (cell types, diseases, conditions) or methodologies. CREdb aims to overcome those limitations by compiling CRE-related information from all major data sources and provides a one-stop-shop repository.

We plan to continuously improve and enhance CREdb in the future. As new technologies linking CREs to their target genes such as activity-by-contact (ABC) measurements become more widely applied, we will regularly update new ABC results/predictions as they become available for more cell types and thereby augment CREdb’s ability to link putative CREs to their target genes in a context dependent manner.

## Methods

### Database design

**Fig. 4:**
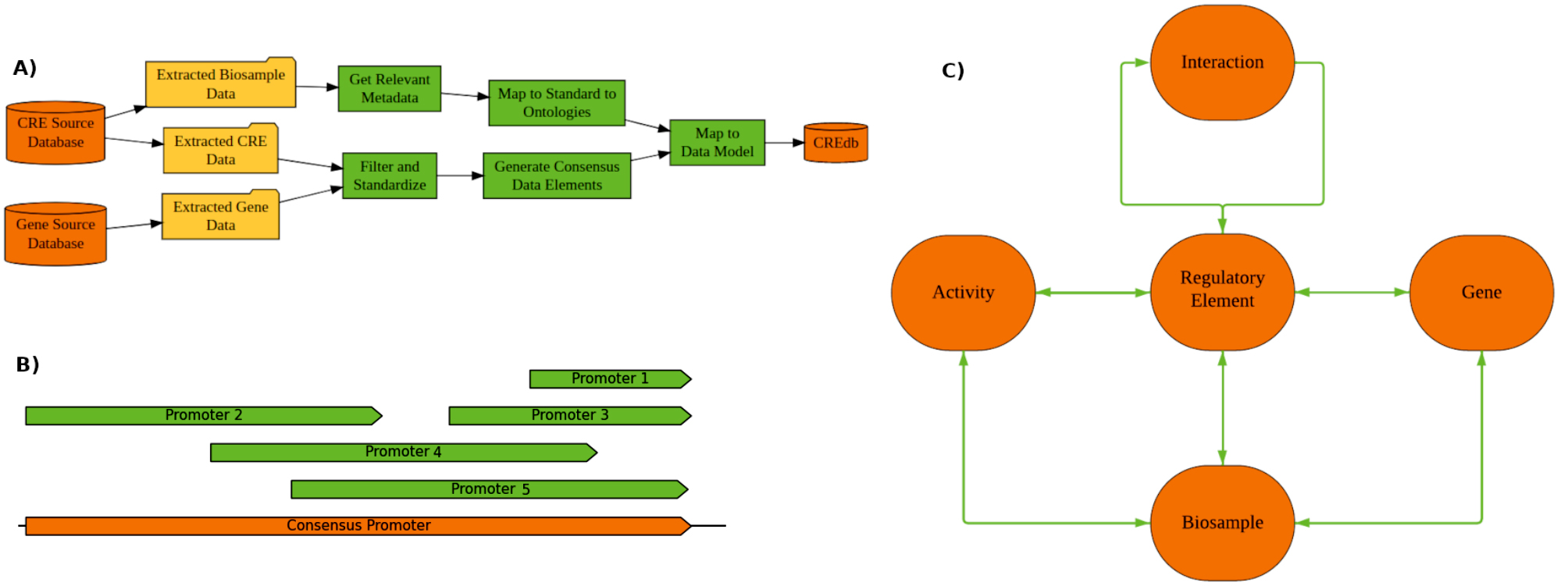
Methodology and Structure of CREdb **(A)** Curation methodology for the generation of CREdb. Raw data is extracted from databases containing data pertaining to CREs, biosamples, and genes. Relevant metadata is then collected for the biosamples and these samples are mapped to standard ontologies to facilitate efficient comparisons between source datasets. CREs and genes are filtered to only human sources and standardized to the same terminology and, where necessary, reference genome. Consensus data elements are then generated based on overlap between each group. Curated CREs, genes, and biosamples are then mapped to the data model to generate the final CREdb. **(B)** Consensus generation of elements. For each element type (promoter, enhancer, etc.), elements were clustered where they had at minimum 20% overlap and condensed into a consensus range for the element. In this example, five promoters have sufficient overlap to be considered a part of a single consensus element. This allows for more sensitive detection of the element when querying with data that might not match any one variant of the site identified in the source databases. **(C)** Data model of final CREdb resource. This conceptual model represents the entities and relationships of the final CREdb. Regulatory elements sit at the center of the model, with interactions between themselves (enhancer/promoter interactions). For each biosample, relationships are identified between genes, regulatory elements, and their respective activity in that sample

A data model was designed to store information related to regulatory elements including promoters, enhancers, silencers, transcription factors, and called segments (other elements). In addition to these elements, the data model also stores the genes they interact with, biosamples with data supporting the regulatory elements, biological activity of regulatory elements in these biosamples, and the data sources used to collect these elements. The conceptual form of this data model is presented in Fig. 4C, and detailed information about their attributes is provided in Supplemental Methods.

### Data sources

Regulatory element data with corresponding biospecimen annotations were collected from the following databases for inclusion in CREdb: ENCODE SCREEN [5], ENdb [6], RefSeq [11], SilencerDB [12], Silencer-Candidates [13], ReMap2020 [14], GeneHancer [15], FANTOM5 [16], EpiMap [17], Ensembl Regulatory Build [18] and EnhancerAtlas [19]. Gene data were collected from the following databases for inclusion in CREdb: HGNC [20], Gencode [11], and RefSeq [7]. These databases were downloaded on 22Jul2021.

### Data curation

Data from each database were filtered to only human regulatory elements and genes. Duplicate entries were removed, and available data were mapped to the CREdb database tables. Database-specific choices were made during the curation process to convert the available data into the universal format provided by the data model. For data sources where the elements were aligned to GRCh37, the elements were remapped to GRCh38 to harmonize all elements against the same reference genome. The data sources that required remapping were FANTOM5, EPIMAP, SilencerDB, EnhancerAtlas, SilencerCandidates, and ENdb. Specifics of these choices are specified in Supplemental Methods. A diagram of the curation and mapping process can be found in Fig. 4A.

To harmonize the metadata for biosamples across various data sources, raw values were classified into distinct categories, including “cell line,” “primary cell,” “in vitro differentiated cells,” “tissue” or “disease.” Original biological source values were then mapped to standard ontologies widely accepted by the biomedical community. For example, for “cell line” type, the raw values are mapped to Experimental Factor Ontology (EFO), Cellosaurus (CVCL), and Cell Line Ontology (CLO) terms. For “primary cells” and “in vitro differentiated cells”, most of the values are mapped to the Cell Ontology (CL) standard terms. For the “tissue” and “disease” types, most of the values are mapped to Uber-anatomy ontology (UBERON) and the Human Disease Ontology (DOID), respectively. To enrich the annotations for cell lines and primary cells, additional information about corresponding diseases and tissues were extracted as well.

Standard ontology alignment was generated using a custom Excel plugin based on SciGraph [21], which stores and manages ontologies. The plugin automatically searches for the closest term in a selected ontology and returns the preferred term (PT), Ontology, Ontology ID, and a matching score. Manual review by domain experts was then performed for all terms with a matching score below 0.80 (where 1 is the highest score for the exact match).

### Consensus generation

The harmonized data were used to generate three sets of combined tables utilizing data from all available databases:

- A combined element table generated by concatenation and genomic re-sorting of all element tables, activity tables, and element-gene interaction Table .
- A consensus element table derived from the combined element table using the ‘bedmap’ tool from the ‘bedops’ tool suite [22] to perform clustering by a reciprocal overlap of 20% with all elements of the same type (enhancers, promotors, silencers, and called segments). Transcription factors were excluded as they are a product of ReMap’s clustering, and elements less than 10 bp or greater than 50,000 bp were excluded based on size. An overview of this methodology is presented in Fig. 4B.
- A master activity table was created by taking the consensus at the biosample level by merging within each consensus element the activity profiles of all biosample replicates, then aggregating these across all datasets to determine consensus activity. FANTOM5 and EnhancerAtlas were handled differently, with gene TPM (Transcripts Per Million) aggregated by taking the median across replicates; and the percentile expression calculated by subsequently rank-normalizing within sample. An element was considered active in the master activity table if any of the following were true:
- it had greater than or equal to two datasets where it was called active.
- FANTOM5 and EnhancerAtlas expression percentile both greater than zero.
- FANTOM5 or EnhancerAtlas expression percentile greater than 20%.

### Activity by contact data

Candidate enhancers identified using the Activity by Contact (ABC) model by Nasser et al. [8] were included as a separate table by extracting each candidate and its relevant information from the provided bed files.

### Enrichment of GWAS signals in tissue-specific CREs

GWAS lead variants were downloaded from the GWAS Catalog (https://www.ebi.ac.uk/gwas/api/search/downloads/alternative). A BED file was generated by expanding 100 bps both upstream and downstream of each lead variant. The overlap between GWAS signals BED file and tissue specific consensus regulatory elements were performed using bedtools (version 2.27.1) intersect command. Experimental Factor Ontology (EFO) mapping provided by the GWAS Catalog was used to annotate the trait/phenotype of each GWAS study. The enrichment analysis was carried out with hypergeometric testing with Python library Scipy (v1.7.3). Each unique variant-phenotype pair is counted only once. Phenotypes with fewer than ten hits in a tissue were excluded from subsequent analysis.

### snATAC-seq analysis

The snATAC-seq dataset was downloaded from GEO (https://www.ncbi.nlm.nih.gov/geo/query/acc.cgi?acc=GSM6459701) in the form of fragment alignment genomic coordinates (GSM6459701_NOSN01_snATAC.fragments.tsv.gz) and peak by cell count matrix (GSM6459701_NOSN01_snATAC.filtered_peak_bc_matrix.h5). Signac R package (version 1.11.0) was used to load the downloaded dataset and perform downstream analysis. LSI dimensionality reduction was carried out by implementing term frequency-inverse document frequency (TF-IDF) transformation followed by singular value decomposition (SVD). The top 30 LSI projections (except the first one) were used to compute Shared Nearest Neighbors (SNNs), which were then used to cluster nuclei based on the SLM algorithm in the FindClusters function. Gene activity scores were computed for protein-coding genes by summing snATAC-seq reads mapped in the gene body and the promoter (5 kb upstream to TSS) by using GeneActivity function. Cell clusters were annotated by inspecting gene activity scores of known marker genes of neural cell types. The genomic coordinates of smooth muscle cell specific CREs were lifted over to hg19 using the liftOver command in the rtracklayer R package (version 1.60.1) to be compatible with the snATAC-seq fragment coordinates.

### Electronic supplementary material

Below is the link to the electronic supplementary material.

Supplementary Material 1

## Data Availability

The datasets generated as part of the CREdb are available in the Zenodo repository (https://zenodo.org/records/11407329, doi: 10.5281/zenodo.11407329).
