# Supplementary material for: CREdb: A comprehensive database of Cis-Regulatory Elements and their activity in human cells and tissues
Source: Epigenetics Chromatin. 2024 Jul 16;17:21. doi: 10.1186/s13072-024-00545-7 (PMC11253421; doi:10.1186/s13072-024-00545-7)
Supplement: Supplementary file 1 — Supplementary Material 1 [file 13072_2024_545_MOESM1_ESM.docx]

Supplemental Methods:

ENCODE: The type of element was converted to the univeral definitions in the database as follows: pELS and dELS were defined as enhancers, PLS were defined as promotors, Low-Dnase were defined as inactive, and all others were defined as called segments. The activity level was defined as follows: pELS, dELS, and PLS were defined as “active”, High-H3K27ac, High-H3k4me3, High-H3K4me3, High-H3K2ac were defined as “partial-active”, Low-Dnase and CTCF-only, CTCF-bound were defined as “inactive”. All other elements were assigned “unclassified” for activity.

ENdb: Activity was determined by presence, any enhancer tagged with a biological source was treated as an assertion of activity within the sample. Unlike the enhancers themselves, the enhancer gene interactions were not directly validated in this database, so were not included in the database.

EnhancerAtlas: Activity for a sample was considered “active” if the percentile rank was greater than zero.

Ensembl Regulatory Build: Elements were mapped to types by their “Name” variable in the database. “enhancer” elements were mapped as Enhancers, “promotor” elements as Promoters, “TF_binding_site” elements as TFBS, and “promoter_flanking_region”, “open_chromatin_region”, and “CTCF_binding_site” as Called Segments. All other name values were ignored. Transcription factor binding sites in this database do not reference the transcription factor that binds. Because of this, the cluster was set to reference the Ensembl transcription factor binding site, the locus references the cluster and a gene “UNKOWN/RETIRED”, and TFBS references the locus and the biosample the binding site was identified in.

EpiMAP: An ‘active window’ of ChromHMM segments is maintained, consisting of all loci that could potentially overlap an element. Each element of the master file (‘master element’ - an enhancer or promoter) is advanced, and all ChromHMM segments ending before the start of the new element are subsequently dropped. Simultaneously, the .bed files for enhancer and promoter calls for the corresponding samples are advanced to the first element that does not end before the start of the master element (‘sample element’). The element and the active segments are intersected to determine the number of bases of the element falling into each ChromHMM state; and the state with the highest number of bases is assigned as the ‘active_state’ for the element within the sample. If the element itself originates from that sample (I.e., the master element matches the sample element) then ‘called_active’ is set to TRUE.

Simultaneously, the correlation-based enhancer-gene interaction tsv file in links/links_corr_only (corresponding to the same sample as the ChromHMM segmentation file) is advanced, and where the master element matches the element of the interaction file, one or more enhancer-gene interactions will be added to the EGI table. Interestingly, the links.tsv provides an enhancer state (i.e., ‘E10’) in addition to the gene and confidence score. This state does not always match the state of the enhancer in the chromatin segmentation file. For instance, the enhancer at chr1:8950899–8951040 in sample BSS01208 is linked to ENSG0000074800 with the state ‘E10’ (ChromHMM state 10, ENHA1). However, the chromatin segmentation for this sample has chr1:8950600–8951200 (a segment that both starts before and ends after the called enhancer) in state ‘EnhA2’ (or ‘E11’). We can find no information regarding such a discrepancy. We have retained all such elements for completeness.
ENCODE

Raw metadata for ENCODE was provided (automatically) with the bulk download of segmentation files and is available in raw-data/metadata_ENCODE.tsv and were provided to the curation team for term mapping and annotation.

The file “V2.hg38-cCREs-Final.bed.gz” was obtained from github.com/weng-lab/ENCODE-cCREs/blob/master/Version-2/cCRE-Pipeline/Annotations/V2.hg38-cCREs-Final.bed.gz (also available as a track from the UCSC genome browser) and used as a “master element” file, and the type of the element was defined as follows:

pELS : enhancer

dELS : enhancer

PLS : promoter

Low-DNase : inactive

<all others> : called_segment

and database identifiers were assigned to all master elements. Although all elements are present in the master table, their activities are not fully summarized. For instance, the first element:

chr1 181251 181601 EH3D2115304 EH38E1310153 pELS,CTCF-bound

appears in many samples with various states (Unclassified in ENCFF641KGL, DNAse-only in ENCFF706VBJ, Low-DNAse in ENCFF507EZK, pELS in ENCFF036NSJ and ENCFF891NBH, etc.). Sample-level activity profiles are determined by applying the following logic to each sample-specific ENCODE .bed file:

The bed file position (chr, start, stop) is used to assign the element ID via an exact match

The activity type is assigned by:

pELS : active

dELS : active

PLS : active

High-H3K27ac : partial-active

High-H3K4me3 : partial-active

High-H3K4me3,High-H3K27ac : partial-active

Low-DNase : inactive

CTCF-only,CTCF-bound : inactive

High-CTCF : unclassified

DNAse-only : unclassified

Unclassified : unclassified

“called_active” is set if the activity type is “active”

“is_ctcf_bound” is set if the state has “,CTCF-bound"

EnDB

Sample metadata for EnDB was obtained from the EnDB website and is available in raw-data/ENDB/ENDB_biosample_meta.csv. This data was provided to the curation team for term mapping and annotation.

The enhancer file from the EnDB website was subset to those elements tagged with Species=’Human’, and duplicate enhancers (if any) were dropped. The elements were sorted genomically and mapped from gene symbol to the database gene ID using the ‘symbol’ column of the genes table.

Activities were simply determined by presence. Any enhancer tagged with a biological source in the enhancer file is treated as an assertion of activity within this sample, and that (enhancer, biological sample) pair appears in the enhancer_activity table with called_active=’True’.

The enhancer table was converted into a .bed file, and lifted from hg19 to b38 coordinates, and converted back to compressed csv.

The ‘target_gene’ column of the EnDB table is used to define 427 enhancer-gene interactions. However, unlike the enhancers themselves (which are experimentally validated), some of these interactions in many cases are not themselves directly validated (via e.g., CRISPR) and many are determined only based on proximity. As such this table is included in transformed-data/EnDB/enhancer_gene_interaction.csv.gz but is not incorporated in the combined EGI table.

EnhancerAtlas

EnhancerAtlas data was downloaded for humans from the EnhancerAtlas website. No metadata on these samples is available, so the column names of ‘matrix_hs.csv’ were provided to the curation team for mapping and annotation.

The first column of the homo sapiens activity matrix was used to define the chromosome, start, end, and strand of the enhancers, generating the enhancer table. The columns of the table define the activity scores for all biosamples; and these scores were additionally transformed to percentile expression via taking the rank (scaled to [0, 100]) within each column, breaking ties by the row-order of the elements. Additionally, wherever the raw value of the activity score was 0, the corresponding percentile rank was also set to 0. An enhancer was defined as active if the percentile rank was greater than 0.

The various enhancer-promoter tables provided on the EnhancerAtlas website were used to define enhancer-gene pairs. The first column of these tables was used to look up the enhancer (by location), as well as the gene (via ensembl ID). The second column defines a ‘confidence score’ which is incorporated in the transformed data but dropped for the purposes of merging with other datasets.

The enhancer table was converted to a .bed file, lifted from hg19 to b38, and converted back to csv.

Ensembl Regulatory Build

GFF files for the Ensembl regulatory build were downloaded from the Ensembl FTP site. Metadata was identified by crawling the Ensembl FTP site for files with a large number of text matches to the GFF file IDs, yielding a relevant metadata file at ftp.ensembl.org/pub/release-104/mysql/homo_sapiens_funcgen_104_38/epigenome.txt.gz. The names of the GFF files, along with the identified putative metadata file, were provided to the curation team for mapping and annotation.

Each GFF file was processed separately. Element types were segregated based on the Name column, with the following definitions:

Enhancer : Name = ‘enhancer’

Promoter : Name = ‘promoter’

TFBS : Name = ‘TF_binding_site’

Called Segment : Name in ‘promoter_flanking_region’, ‘open_chromatin_region’, ‘CTCF_binding_site’

All other Name values are ignored. The ‘activity’ field is propagated into the ‘activity’ field of the corresponding element record, and the record has ‘called_active = True’ where ‘activity = ACTIVE’. Element positions were taken directly from the GFF, and all strands were set to unstranded.

During processing, all elements are stored in a table mapping “chromosome:start-end” to “element ID” s. Any re-occurrence of a position re-uses the original ID assigned to that position (indeed, each GFF contains the same elements with differing activity annotations).

The transcription factor binding sites, while relevant to a biosample, do not provide the actual transcription factor that binds. Therefore, the cluster, locus, and site tables were arranged as:

Cluster – references the location of the ENSEMBL TF binding site

Locus – references the above cluster, and a special UNKNOWN/RETIRED gene

TFBS – references the above locus, and the biosample corresponding to the GFF

EpiMAP

EpiMap data was obtained from ftp://personal.broadinstitute.org/cbiox/epimap; and the files metadata/biosample_encode_accession_mapping.tsv and metadata/main_metadata_table.tsv were merged and provided to the curation team for mapping and annotation.

The individual bed files in mark_matrices/enhancers_bysample/ and mark_matrices/promoters_bysample/ were concatenated and sorted karyotypically using bedtools, forming a master list of enhancers and promoters. Then, the segmentation files in ChromHMM/observed_aux_18_hg19/CALLS (as the mark_matrices bed files are provided in hg19 coordinates) are each processed in turn as follows:

An ‘active window’ of ChromHMM segments is maintained, consisting of all loci that could potentially overlap an element. Each element of the master file (‘master element’ - an enhancer or promoter) is advanced, and all ChromHMM segments ending before the start of the new element are subsequently dropped. Simultaneously, the .bed files for enhancer and promoter calls for the corresponding samples are advanced to the first element that does not end before the start of the master element (‘sample element’). The element and the active segments are intersected to determine the number of bases of the element falling into each ChromHMM state; and the state with the highest number of bases is assigned as the ‘active_state’ for the element within the sample. If the element itself originates from that sample (I.e., the master element matches the sample element) then ‘called_active’ is set to TRUE.

Simultaneously, the correlation-based enhancer-gene interaction tsv file in links/links_corr_only (corresponding to the same sample as the ChromHMM segmentation file) is advanced, and where the master element matches the element of the interaction file, one or more enhancer-gene interactions will be added to the EGI table. Interestingly, the links.tsv provides an enhancer state (i.e., ‘E10’) in addition to the gene and confidence score. This state does not always match the state of the enhancer in the chromatin segmentation file. For instance, the enhancer at chr1:8950899–8951040 in sample BSS01208 is linked to ENSG0000074800 with the state ‘E10’ (ChromHMM state 10, ENHA1). However, the chromatin segmentation for this sample has chr1:8950600–8951200 (a segment that both starts before and ends after the called enhancer) in state ‘EnhA2’ (or ‘E11’). We can find no information regarding such a discrepancy. We have retained all such elements for completeness.

The result of this process are tables for enhancers, enhancer activities, promoters, promoter activities, and enhancer gene interactions. The enhancer and promoter activity tables are reformatted into .bed files, lifted from hg19 to b38, and converted back to .csv files.

FANTOM5

The FANTOM5 (v9 – b38-aligned) annotated TPM matrix was processed as follows to produce promoter and enhancer tables (and their respective activities). First, the header of the table was parsed to provide a mapping between column names and biosample IDs (from the FANTOM5 metadata). The sample ‘middle temporal gyrus : donor10252 : CNhs12316’ is mapped to ‘medial temporal gyrus’ (which appears in the metadata, while ‘middle temporal gyrus’ does not). The TPM matrix was read in, and split into promoters (annotation of the form “p[0–9]+@[A-Za-z0-9]+”, where the value after “@” is the gene symbol) and enhancers (annotation of the form “hg_[0–9]+”). There are ambiguous entries with the latter format that are nonetheless annotated with transcript IDs – these are treaded as enhancers rather than promoters.

Coordinates for these IDs are provided by FANTOM5 in separate “fair + CAGE” and “liftover + CAGE” bed files; and these coordinates are used in defining enhancer and promoter records. Each row annotation is converted into a promoter or enhancer record (depending on the format). The TPM matrices for enhancers and promoters are rank-transformed (within sample) and divided by the maximum rank to produce values in [0–1]. The rank value associated with TPM = 0 is then set to 0; and the percentile rank values are then multiplied by 100 and cast to integers. Each value (corresponding to the activity of one element in one sample) becomes the basis for enhancer activity or promoter activity records. ‘called_active’ was defined as a rank percentile > 0.

Enhancer-promoter interactions from SlideBase were parsed to link between enhancer IDs and promoter IDs (this is a many-to-many mapping). As SlideBase was based on a previous release of FANTOM5 and a subset of the samples, not all elements in SlideBase are present in FANTOM5v9. These associations (and associated confidence scores for tissues and cell types) are incorporated into the enhancer-promoter interaction table.

Genehancer

Because the available export of Genehancer (free version accessible on UCSC) does not provide tissue or cell-line information for individual elements, all Genehancer elements are associated with the UNIVERSAL/UNSPECIFIED placeholder sample.

The Genehancer bed file splits elements into ‘enhancer’, ‘promoter’ and ‘enhancer/promoter’ types. For this database, ‘enhancer/promoter’-type elements are assigned either to ‘enhancer’ or ‘promoter’ based on their nearest gene: <=25bp are assigned to ‘promoter’, and > 25bp to ‘enhancer’.

The Genehancer files on UCSC also contain interaction files (see PMC5467550 for methodology), which are used to associate both enhancers and promoters with target genes. For promoters, this association is incorporated into the promoter table, while for enhancers these associations are transformed into records in the enhancer/gene interaction table. ‘called_active’ is always set to true.

RefSeq

The RefSeq bed file (obtained from the UCSC genome browser website) was split into element types using the ‘name’ field, with ‘enhancer’, ‘promoter’, and ‘silencer’ mapping to themselves, any name containing the substring ‘protein_bind’ mapping to a transcription factor cluster/locus, and ‘insulator’ or ‘enhancer_blocking_element’ or ‘transcriptional_cis_regulatory_region’ mapping to ‘called_segment’.

The corresponding regular expressions were used to extract biological sample information from the free text ‘function’, ‘_mouseOver’, and ‘experiment’ fields:

parsers_regex = {

'enhancer': ['.*enhancer in (.*)', '.*enhancer in: (.*)', '.*in (.*) cells.*', 'enhancer in: (.*)'],

'promoter': ['.*promoter in (.*) cells.*', '.*(.*) cell-specific.*', '.* activity in (.*) cells.*',

'.*drives expression in (.*)', '.*promotes expression in (.*)',

'.*drives expression of transgene in (.*)', '.*core promoter in (.*) cells'],

'transcriptional_cis_regulatory_region': ['expression in (.*)'],

'protein_bind': [],

'enhancer_blocking_element': ['.*in (.*) cells'],

'silencer': ['.*in (.*) cells'],

'insulator': ['.* in (.*) cells']

}

Any matching values were subsequently post-processed to split multiple-biosample experiments into responding cells (e.g., enhancer in X and Y) and non-responding cells (e.g., promotes expression in X cells but not in Y). Records for which the regular expressions had no match were linked to the UNKNOWN/REVOKED biological source. ‘called_active’ was set to True for all biological samples parsed through the above process.

Transcription factor binding sites were structured as followed: Each TFBS entry is assigned to a unique tf_cluster record (I.e., tf_locus and tf_cluster are in 1-to-1 correspondence for RefSeq) Any gene referenced in the gene_id or free-text fields as the binding gene is assigned to the tf_locus record – and if no such gene was present then an UNKNOWN catch-all gene is assigned. Each of the samples parsed from the free-text fields generate a tf_binding_site record, with called_active = True. This record references both the biological sample ID and the tf_locus ID.

REMAP2020

The REMAP2020 ‘crm’ (cis-regulatory modules) files define the transcription factor clusters, while the non-redundant tf file ‘nr_macs2’ defines both the tf_locus and tf_binding_site records. The CRM file is generated (by REMAP) through clustering the non-redundant transcription factor bed records.

The crm and nr_macs2 files are simultaneously iterated, which each crm entry generating a unique tf_cluster record, and all overlapping nr_macs2 records generating at least one record as follows:

If the referenced transcription factor and location are new for the cluster, create a tf_locus record bridging the cluster and transcription factor, and referencing the midpoint of the location

If the referenced transcription factor and location are not new, retrieve the existing tf_locus record

Create a tf_binding_site record referencing the above tf_locus ID (from (1) or (2)) and the biological sample present in the nr_macs2 bed record with called_active = True

For a small number of experiments, there are multiple transcription factors that were used as part of a pooled ChIP-seq experiment. In these cases, rather than associating the locus with a transcription factor directly, a ‘tf_pool’ record is created for the experiment, associating a unique pool identifier with multiple transcription factors. The tf_locus record then references that ‘tf_pool’ identifier (in tf_pool_id), while leaving transcription_factor empty.

Silencer-Candidates

As a very large and ragged collection of intervals, Silencer-Candidates was initially converted into a set of consensus intervals as follows:

ENCODE and ROADMAP element predictions were combined and sorted

Elements were clustered with a slack (minimal overlap) of 50bp

Singleton elements were left as-is

Cluster consensus intervals were determined as the 20th percentile of the start position, and 80th percentile of the end position, across all intervals in the cluster.

These consensus clusters were then used to generate Silencer-Candidates records as follows:

Clusters consisting only of elements not predicted to be silencers (I.e., non-candidates) were dropped

Original positions were saved as ‘sample_speicific_start’ and ‘sample_specific_end’, and set to reference consensus positions and IDs (for members of non-singleton clusters)

‘is_active’ is set to True where the SVM prediction is ‘Candidate_Silencer’

Silencer positions and IDs were written to the silencer table, while sample-specific positions and activities were written to the activity table.

The silencer table (with elements) is converted to .bed format, lifted to b38, and re-converted to csv.

SilencerDB

SilencerDB is provided as a single long-form bed file with positions and biological samples. Unique positions are saved as silencer records, while biological sample information for a position is saved to the activity table with ‘called_active’ set to True.

The resulting csv file of unique silencer records is converted to .bed format, lifted to b38, and re-converted to csv.

Biological Source Metadata Curation

The aggregated metadata from various data sources in the above steps provides information about the biological “Source Value” s of the regulatory elements and their corresponding “Source Dataset” and “Source File Name”. Additional metadata is scarpered from source webpages (e.g., “Enhancer Atlas”) or REST API (e.g., “ENCODE”, “Remap” and “Epimap”) to help us cross-reference and annotate the source values. To annotate the metadata for the biological sources across various data sources, we classified the source values according to the value types, including “cell line”, “primary cell”, “in vitro differentiated cells”, “tissue” or “disease”. Biological samples from organisms other than humans are excluded from further processing based on scope of this project.

To harmonize the metadata for biosamples across various data sources, we classified the raw values into different categories, including “cell line”, “primary cell”, “in vitro differentiated cells”, “tissue” or “disease”. We then harmonized the original biological source value across data sources using standard ontologies that are widely accepted by the biomedical community. For example, for “cell line” type, the raw values are mapped to Experimental Factor Ontology (EFO), Cellosaurus (CVCL), and Cell Line Ontology (CLO) terms. For “primary cells” type, most of the values are mapped to the Cell Ontology (CL) standard terms. For the “tissue” and “disease” types, the majority of the values are mapped to Uber-anatomy ontology (UBERON) and the Human Disease Ontology (DOID), respectively. To enrich the annotations for cell lines and primary cells, additional information about corresponding diseases and tissues were extracted as well.

Standard ontology alignment was generated using a custom Excel plugin previously created by Rancho Biosciences. The plugin is based on an open-source platform called SciGraph, which stores and manages ontologies. The plugin automatically searches for the closest term in a selected ontology and returns the preferred term (PT), Ontology, Ontology ID, and a matching score. Manual review by domain experts was then followed for all terms with a matching score below 0.80 (where 1 is the highest score for the exact match).

For quality assurance (QC), at least 10% of the mappings are reviewed by two separate curation scientists. The curators verify the unique mapping of standard terms and corresponding IDs and check the consistency of annotations across various data sources. All unclear annotations (if the source data does not provide enough information to enable accurate curation) are marked as “yes” in a separate column called “Unclear_Annotation” (The curator’s comments on unclear annotations are in the “Curator_Comments” column).
